# Correlations between Cytokine Levels, Liver Function Markers, and Neuropilin-1 Expression in Patients with COVID-19

**DOI:** 10.3390/vaccines10101636

**Published:** 2022-09-29

**Authors:** Salma A. El Kazafy, Yasser M. Fouad, Azza F. Said, Hebatallah H. Assal, Tarek M. Ali, Amr E. Ahmed, Basem H. Elesawy, Osama M. Ahmed

**Affiliations:** 1 Biotechnology Department, Faculty of Postgraduate Studies for Advanced Sciences, Beni-Suef University, Beni-Suef 62521, Egypt; 2 Department of Internal Medicine, Faculty of Medicine, Minia University, Minia 61519, Egypt; 3 Department of Pulmonary Medicine, Faculty of Medicine, Minia University, Minia 61519, Egypt; 4 Department of Chest Medicine, Faculty of Medicine, Cairo University, Cairo 11562, Egypt; 5 Department of Physiology, College of Medicine, Taif University, P.O. Box 11099, Taif 21944, Saudi Arabia; 6 Department of Pathology, College of Medicine, Taif University, P.O. Box 11099, Taif 21944, Saudi Arabia; 7 Physiology Division, Zoology Department, Faculty of Science, Beni-Suef University, Beni-Suef 62521, Egypt

**Keywords:** cytokines, liver, NRP-1, NF-κB, COVID-19, moderate, severe

## Abstract

**Aim:** The study evaluated the correlations between cytokine levels, liver function markers, and neuropilin-1 (NRP-1) expression in patients with COVID-19 in Egypt. The study also aimed to evaluate the accuracy sensitivity, specificity, and area under the curve (AUC) of the tested laboratory parameters in identifying COVID-19 infection and its severity. **Patients and Methods:** Fifty healthy subjects and 100 confirmed patients with COVID-19 were included in this study. COVID-19 patients were separated into two groups based on the severity of their symptoms. Serum ALT, AST, albumin, C-reactive protein (CRP), interleukin (IL)-1β, IL-4, IL-6, IL-18, IL-35, prostaglandin E2 (PGE2), and thromboxane A2 (TXA2) were estimated. We measured the gene expression for nuclear factor-kappa B p50 (NF-κB p50) and nuclear factor-kappa B p65 (NF-κB p65) and NRP-1 in blood samples using quantitative real-time polymerase chain reaction (qRT-PCR). AUC and sensitivity and specificity for cytokine levels and NF-κB p50 and NF-κB p65 and NRP-1 in identifying COVID-19 infection were also determined in both moderate and severe patient groups using receiver-operating characteristic curve (ROC) analysis. **Results:** All patients with COVID-19 showed higher serum activities of liver enzymes, levels of CRP, IL-1β, IL-4, IL-6, IL-18, IL-35 PGE2, and TXA2, and mRNA expression of NF-κB p50, NF-κB p65, and NRP-1 than healthy subjects. The severe group exhibited a significant increase in serum ALT, AST and IL-6 and a significant decrease in albumin, IL-1β, TXA2, and NF-κB p65 levels compared to the moderate group. In all patients (moderate and severe), all cytokines were positively correlated with NF-κB p50, NF-κB p65 and NRP-1 expression levels. Serum ALT and AST were positively correlated with CRP, cytokines (IL-4, IL-6, IL-18, IL-35 and TXA2), and NF-κB p50 and NF-κB p65 expression levels in both moderate and severe groups. They were also positively correlated with serum IL-1β level in the severe COVID-19 patient group and with NRP-1 expression in the moderate group. Using the logistic regression analysis, the most important four statistically significant predictors associated with COVID-19 infection in the study were found to be IL-6, TAX2, NF-κB p50 and NF-κB p65. ROC analysis of these variables revealed that three of them had AUC > 0.8. In moderate cases, AUC of the serum TXA2 level and NF-κB p65 expression were 0.843 (95% CI 0.517–0.742, *p* < 0.001) and 0.806 (95% CI 0.739–0.874, *p* < 0.001), respectively. In the severe group, AUC of serum IL-6 level was 0.844 (95% CI 0.783–0.904, *p* < 0.001)**.** Moreover, Il-6 had a sensitivity of 100% in both moderate and severe groups. **Conclusions**: This study concluded that liver injury in patients with COVID-19 may be strongly attributed to the cytokines storm, especially IL-6, which was positively correlated to NF-κB p50, NF-κB p65 and NRP-1 mRNA expression levels. Moreover, ROC analysis revealed that IL-6, TXA2, and NF-κB p65 could be useful in predicting the possibility of infection with COVID-19, and IL-6 could be of possible significance as a good predictor of the severity and disease progress. However, RT-qPCR for SARS-CoV-2 detection is essential to confirm infection and further clinical studies are required to confirm this elucidation.

## 1. Introduction

The current coronavirus disease 2019 (COVID-19) pandemic is caused by a new severe acute respiratory syndrome coronavirus 2 (SARS-CoV-2). It has posed significant problems due to its rapid spread and many clinical consequences [1]. COVID-19 has spread to over 225 nations, resulting in almost 140 million confirmed illnesses and 3 million fatalities as of 15 April 2021 [2]. The pathogenesis of SARS-CoV-2 begins when virus particles connect to the angiotensin-converting enzyme 2 (ACE2) receptors on the surface of endothelial cells in the lungs, causing macrophages, natural killer cells, and other immune cells to produce chemokines and cytokines [3]. These dysregulated and hyper-inflammatory actions eventually lead to inflated levels of the cytokine concentrations and can be considered the main reason for multiple organ damage [4]. Prostaglandins (PGs) and their precursors may alter SARS-CoV-2 attachment by modifying ACE2 and transmembrane serine protease 2 (TMPRSS2) expression, as well as virus endocytosis, by controlling lipid vesicle fusions, according to Robb et al. [5]. PGs have the potential to influence the immunological and inflammatory responses elicited by viral pathogens in the host. Both innate and adaptive immunological responses can be aided or hindered by PGs, particularly PGE2. PGs can inhibit cytotoxic T cell responses, type I interferon (IFN) signaling, neutrophil extracellular trap formation, inflammasome activation, and the generation of inflammatory cytokines, in this way. They may also have a role in inflammatory T helper 17 (Th17) responses, nuclear factor kappa B (NF-κB) activation, and the generation of inflammatory cytokines. The strength (e.g., viral load) and timing of stimuli, organ locations, and responding cell types, all play a role in the effects of PGs. Given the importance of cytokine storms in COVID-19 immunopathology and the context-dependent regulation of cytokine production by PGs, it is critical to understand the main cellular sources of cytokines in the lung and peripheral blood, as well as the key stimuli (e.g., SARS-CoV proteins or peptides) and the kinetics of cytokine secretion. NF-κB is a protein transcription factor that has been around for a long time [6], and is a natural immune regulator [7]. In the inner matrix of the mitochondrion, Cogswell et al. [8] observed NF-κB p50 and p65 subunits as well as inhibitory-κBa (IκBa). Inducers of NF-κB include bacterial lipopolysaccharides, ionizing radiation, reactive oxygen species (ROS), cytokines such as tumor necrosis factor-alpha (TNF-α), and interleukin (IL)-1beta (IL-1β), as well as viral DNA and RNA [9]. Activated NF-κB transcription factors increase the expression of a wide range of cytokines (e.g., IL-1, IL-2, IL-6, IL-12, TNF-α, IL-8, lymphotoxin-α (LT-α), and lymphotoxin-β (LT-β)), as well as chemokines (e.g., macrophage inflammatory protein 1 [MIP-1], monocyte chemoattractant protein-1 (MCP1), RANTES, and eotaxin). The intercellular adhesion molecule (ICAM), vascular cell adhesion molecule (VCAM), and E-selectin are examples of adhesion molecules, while acute phase proteins such as serum Amyloid A (SAA) and inducible effector enzymes are examples of acute phase proteins (e.g., inducible nitric oxide synthase; iNOS and cyclooxygenase-2; COX-2). An enhanced level of pro-inflammatory factors, such as IL-2, IL-1, IL-6, IFN-γ, MIP1α, MCP1, and TNF-α has been found in most severe cases since the inception of COVID 19 [10]. The “cytokine storm” found in some COVID-19 patients is caused by invading phagocytic cells such as monocytes and macrophages. During a SARS-CoV-2 infection, intracellular signaling pathways, such as mitogen-activated protein kinase (MAPK) and NF-κB, must be active in order for cytokines to be produced. The majority of these mediators are biologically independent and have pleiotropic downstream effects. SARS-CoV-2 stimulates the p38 MAPK and NF-κB pathways, as does Middle East respiratory syndrome coronavirus (MERS-CoV) and severe acute respiratory syndrome coronavirus 1 (SARS-CoV-1) [11]. The increased production and release of pro-inflammatory cytokines via these signaling pathways harms airway epithelial cells and alveolar tissues, resulting in reduced breathing, acute lung injuries, and acute respiratory distress syndrome (ARDS) [12].

Neuropilin-1 (NRP-1), a member of a family of signaling proteins, has been revealed to serve as an entry factor and potentiate SARS-CoV-2 infectivity In Vitro. These cell surface receptors, with their disseminated expressions, are important in angiogenesis, tumor progression, axonal guidance, viral entry, and immune function. NRP-1 is implicated in several aspects of a SARS-CoV-2 infection, including possible spread through the olfactory bulb and into the central nervous system (CNS) and increased NRP-1 RNA expression in lungs of severe COVID-19 [13]. Recently, it was found that impairment of renal and cardiac function biomarkers in serum might be the result of a cytokine storm and an increase in the expression of ACE-2 and NRP-1 in COVID-19 patients [14,15]. Understanding how SARS-CoV-2 enters and spreads throughout human organs is important for developing preventative and therapy measures.

The cornerstone of the best prevention and therapy, particularly in hospitals, is a precise and prompt diagnosis. The primary diagnostic test for COVID-19 is the real time (RT)-polymerase chain reaction (PCR), but its sensitivity varies [16], sometimes reaching a sensitivity of 71% [17]. If a negative result is obtained but there is a high likelihood of infection, fresh RT-PCR tests should be run as a result of this possibility [18]. This results in a delay in diagnosis and treatment options, both of which are necessary for the patient’s survival as well as for preventive quarantine and hospital administration [19]. Despite the recent development of various logistic prediction models, none of them were completely successful when used in clinical practice [20,21,22,23,24]. Although numerous models demonstrated the best area under the curve (AUC), their implementation in everyday clinical practice is complicated. In order to make quick judgments until a final confirmation is achieved, or even to avoid the need for RT-PCR tests to be performed, it would be helpful to develop a straightforward, accurate, and specific biomarker to detect COVID-19 patients. To enable early detection of COVID-19 patients, particularly in emergency rooms, we contribute to the identification of a practical and precise diagnostic biomarkers of COVID-19 disease by prospectively evaluating the levels of five cytokines, prostaglandin E2 (PGE2), thromboxane A2 (TXA2), TXA2, NF-κB p50, NF-κB p65, and NRP-1.

We conducted this study to look into the relationships between cytokine levels, inflammatory mediators, liver function indicators, and NRP-1 expression in COVID-19 patients as well as the possible usefulness of various detected parameters in the foretelling of COVID-19 infection and its progress and severity. 

## 2. Patients and Methods

### 2.1. Study Population

This study comprised 100 COVID-19 patients with a mean age of 61.05 years. Between March and July 2021, all participants were isolated at Misr International Hospital in Cairo, Egypt. A total of fifty healthy controls were also included in the study. The research was conducted in conformity with the Helsinki Declaration and Good Practice Guidelines. The study was approved by the ethics committee of Misr International Hospital, Cairo, Egypt, and the institutional review board of the Ministry of Health, Cairo, Egypt (No. 3-2021/19).

### 2.2. Patients

A cohort of 100 volunteers confirmed to have been COVID-19 patients were included in this study. All patients included in the study had an RT-qPCR positive COVID test. In addition, the clinical symptoms of COVID-19 infection were confirmed by the Physician. The confirmed COVID-19 patients were categorized into two groups, moderate and severe, according to the guidelines for diagnosis and management of COVID-19 released by National Health Commission of China [25] and also according to our previous publications [14,15]. Patients in the group had symptoms such as fever and respiratory tract symptoms and pneumonia manifestation, which were seen in imaging. Patients in the severe group met any of the following: respiratory distress, respiratory rate ≥ 30 breaths/min, SpO2 ≤ 93% at rest, and PaO2/FIO2 ≤ 300. Patients with greater than 50% lesion progression within 24 to 48 h seen in pulmonary imaging were considered severe cases.

Thyroid dysfunction, alcoholism, autoimmune disorders, eczema, chronic respiratory disease, cancer, kidney failure, liver dysfunction, cerebrovascular diseases, ischemic heart disease, pregnancy and lactation, and patients taking immune-modulatory medicines were excluded from the study.

### 2.3. Laboratory Assay

Blood samples were taken when patients began to experience symptoms of the disease and the symptoms of COVID-19 infection were confirmed by the physician and, at the same time, the COVID test was positive. Participants’ blood samples were obtained in plain tubes (4 mL each). Blood in plain tubes was centrifuged for serum isolation after a 30-min incubation period at room temperature. The serum samples were quickly separated, aliquoted and refrigerated at −40 °C until the biochemical tests were completed (ALT, AST and Albumin). ALT and AST activities were determined using Biosystem S.A. (Barcelona, Spain) reagent kits according to the method of Gella et al. [26]. Using a reagent kit acquired from Human Diagnostics (Wiesbaden, Germany), the concentration of serum albumin was measured using the method of Doumas et al. [27].

Semiquantitative determination of CRP in human serum was done using the rapid latex agglutination technique. Serum levels of IL-1, IL-4, IL-6, IL-18, IL-35, PGE2, and TXA2 were measured using a standard sandwich ELISA kit from R&D Systems (Minneapolis, MN, USA), as directed by the manufacturer.

### 2.4. Ribonucleic Acid (RNA) Extraction and Quantitative Real-Time-Polymerase Chain Reaction (RT-PCR) qRT-PCR

Individual blood samples from healthy people and COVID-19 patients were used to isolate total RNA using TRIzol Reagent (MBI Fermentas, St. Leon-Roth, Germany), and cDNA was synthesized using the High-Capacity cDNA Reverse Transcription kit (Invitrogen, Karlsruhe, Germany) according to the manufacturer’s instructions. qRT-PCR was carried out in a 20 μL system having 10 μL of 1x SsoFast EvaGreen Supermix (Bio-Rad, Hercules, CA, USA), 2 μL of cDNA, 6 μL of RNase/DNase-free water, and 500 nM of the primer pair sequences: NF-κB p50, F: 5-CTGGTGATCGTGGAACAGCC-3 and R: 5-CAGAGCCTGCTGTCTTGTCC-3 (XM008950845.3); NF-κB p65, F: 5-ATGCGCTTCCGCTACAAGTG-3 and R: 5-ACAATGGCCACTTGTCGGTG-3 (MN508965.1); NRP-1, F: 5–AACAACGGCTCGGACTGGAAGA-3 and R: 5 –GGTAGATCCTGATGAATCGCGTG-3 (NM001024628); β–actin, F: 5-GGAACGGTGAAGGTGACAGCAG-3 and R: 5-TGTGGACTTGGGAGAGGACTGG-3 (XM004268956.3). The thermal cycler conditions were as follows: 30 s at 95 °C, then 40 cycles of 5 s at 95 °C and 10 s at 60 °C. A melting curve study was used to determine the melting point (rmp) at 65–95 °C for each reaction. The relative amount of mRNA was measured by estimating the period at which the fluorescent signal exceeded an arbitrarily selected threshold towards the middle of the log-linear amplification step. The amplification data was evaluated with the manufacturer’s program and the Livak and Schmittgen techniques [28], with the variables normalized to β-actin.

### 2.5. Statistical Analysis

The obtained data were represented as mean ± standard error of mean (SEM). They were analyzed using SPSS version 20 for Windows (IBM Corp., Armonk, NY, USA, 2011) [29]. All statistical differences between groups were carried out by Duncan’s test for post hoc analysis. Correlation and regression analysis were estimated by the Pearson correlation coefficient that measures the statistical relationship, or association, between two continuous variables. Significance was calculated at three levels *p <* 0.05; *p* < 0.01 and *p* < 0.001. Using the area under the receiver operator characteristic curve (AU-ROC), we calculated the diagnostic accuracy of the most important variables obtained by regression models to predict the possibility of COVID-19 infection and its severity. We choose the AU-ROC as a general indicator because we know that a model is a perfect classifier when the AUC is 1.

## 3. Results

**Figure 1:**
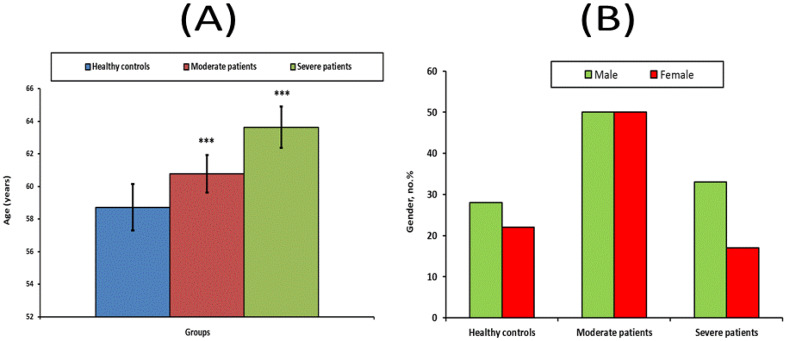
Age (**A**) and gender (**B**) of healthy controls, and moderate and severe groups. *** Significant at *p* < 0.001.

Baseline demographics of all individuals are shown in Figure 1. When comparing the moderate and severe COVID-19 patient groups to the control group, there was a no significant in age (*p >* 0.05). The severe group comprised 66 percent males and 34 percent females (*p* > 0.05), while the moderate group had similar numbers of males and females.

Table 1 shows the levels of serum ALT, AST, albumin, CRP, IL-1β, IL-4, IL-6, IL-18, IL-35, PGE2 and TXA2 levels as well as the expression of NF-κB p50 and NF-κB p65 mRNA in all studied groups. When compared to healthy controls, ALT, AST, albumin, and CRP levels indicated significant increases in moderate and severe COVID (*p <* 0.001). When compared to the moderate group, patients in the severe group had significantly higher ALT (*p <* 0.001), AST (*p <* 0.05), and CRP (*p <* 0.001). In addition, when compared to healthy controls, albumin levels were significantly lower in moderate and severe COVID-19 patients (*p <* 0.001). When compared to the moderate group, albumin levels in the severe group were significantly lower (*p <* 0.01).

In addition, IL-1β, IL-4, IL-6, IL-18, IL-35, PGE2, and TXA2 levels in moderate and severe COVID-19 patients were significantly higher (*p <* 0.001) than in healthy controls. IL-6 was substantially greater (*p <* 0.001) in the severe group than in the moderate group, although IL-1β and TXA2 were significantly higher in the moderate group than in the severe group. When comparing the moderate and severe groups, no significant differences in IL-18, IL-35, or PGE2 were found (*p* > 0.05). 

Furthermore, NF-κB p50 and NF-κB p65 mRNA expression levels in moderate and severe COVID patients were significantly higher (*p <* 0.001) than in healthy controls. In the moderate group, on the other hand, NF-κB p65 mRNA expression had significantly higher levels (*p <* 0.05) than in the severe group. Although it was not significant (*p >* 0.05), the moderate group’s NRP-1 mRNA expression level was higher than the severe group’s. 

Table 1 also shows that NRP-1 mRNA expression levels in moderate and severe COVID patient groups were significantly higher (*p <* 0.001) than healthy controls.

Table 2 illustrates the relationship between CRP, PGE2 and TXA2 levels, and NF-κB p50, NF-κB p65 and NRP-1 mRNA expression with all other tested parameters in the moderate group. CRP, PGE2 and TXA2 levels and NF-κB p50, NF-κB p65 and NRP-1 mRNA expression revealed a significant positive connection with all parameters in the moderate COVID-19 patient group, except for albumin which showed a significant negative correlation (*p* < 0.01) with CRP and a non-significant (*p* > 0.05) negative relationship PGE2, NF-κB p50 and NRP-1 mRNA expression.

Table 3 shows correlations between IL1β, IL4, IL-6, IL-18, and IL-35 with all other tested parameters in the moderate group, where albumin shows a non-significant (*p* > 0.05) relationship with all cytokines except for IL-18 which has a significant negative correlation (*p* < 0.05). IL4, IL-6, IL-18, and IL-35 shows a significant positive correlation with all parameters. IL1β showed non-significant (*p* > 0.05) relationship with ALT, AST and albumin but it had a significant positive correlation (*p* < 0.001) with others.

Table 4 illustrates the relationship between CRP, PGE2, TXA2, NF-κB p50, NF-κB p65 and NRP-1 with all other tested parameters in the severe group. CRP and TXA2 serum levels and NF-κB p50, and NF-κB p65 mRNA expression revealed a significant positive connection with all other parameters in the severe COVID-19 patient group. Serum albumin level showed a significant (*p* < 0.001) negative relationship with CRP, PGE2, TXA2, NF-κB p50, NF-κB p65 and NRP-1 mRNA expression. PGE2 showed non-significant (*p* > 0.05) relationship with AST, while NRP-1 mRNA expression showed non-significant (*p* > 0.05) relationship with ALT and AST.

Table 5 shows the correlation between serum IL1β, IL4, IL-6, IL-18, and IL-35 levels with all other tested parameters in the severe group. All serum cytokine levels revealed a significant positive connection with all parameters in the severe COVID-19 patient group, except for albumin, which showed a significant negative relationship with all cytokines and NF-κB p50 expression, which had non-significant (*p* > 0.05) positive relationship with serum IL-4 level.

**Figure 2:**
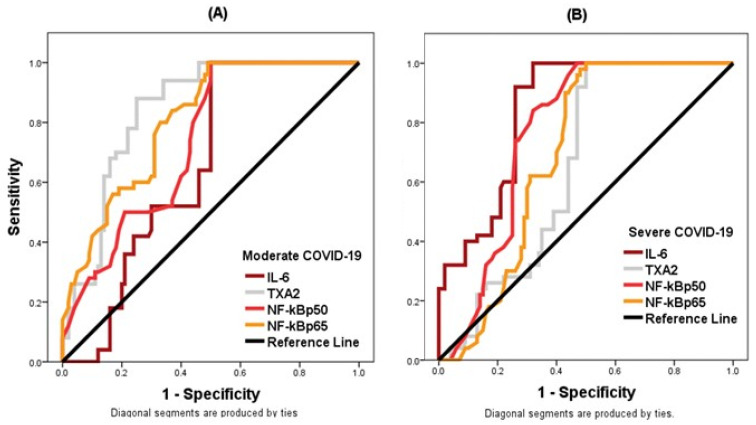
ROC curves of laboratory biomarkers as predictors of disease in the moderate group (**A**) and in the severe group (**B**).

The results of ROC analysis carried out for the most important variables (IL-6, TXA2, NF-κB p50, and NF-κB p65) obtained by entering the data into logistic regression models are represented in Table 6 for the moderate group and Table 7 for the severe group, and in Figure 2 for both moderate and severe groups. 

In the ROC study, a total of four parameters produced a significant result in both moderate (Table 6) and severe (Table 7) groups. In the moderate group of COVID-19 patients, ROC analysis indicated that AUC were 0.656, 0.843, 0.735, 0.806 for IL-6, TXA2, NF-κB p50 and NF-κB p65 with sensitivities of 100, 88, 80, and 80%, and with specificities of 50, 75, 56, and 67% respectively. Only TXA2 and NF-κB p65 had AUC higher than 0.8 (Table 6 and Figure 2). On the other hand, ROC analysis for the same four variables in the severe group of COVID-19 patients revealed that AUC were 0.844, 0.657, 0.765, 0.694 for IL-6, TXA2, NF-κB p50 and NF-κB p65 with sensitivities 100, 92, 74, and 70% and specificities of 68, 53, 74, and 60 %, respectively. Thus, only IL-6 showed an AUC higher than 0.8 in the severe group (Table 7 and Figure 2). 

## 4. Discussion

In the present study, IL-1β, IL-4, IL-6, IL-18 and NRP-1 mRNA expression levels were positively correlated with IL-35, PGE2, TXA2, NF-κB p50, NF-κB p65 mRNA expression levels. Understanding the method by which SARS-CoV-2 enters and spreads throughout human organs is crucial for designing anti-viral strategies. ACE2 is used as a host receptor by SARS-CoV-1 and SARS-CoV-2 [30]. Furthermore, Cantuti-Castelvetri [31] discovered that the cellular receptor NRP-1, which is known to bind furin-cleaved substrates, enhances the infectivity of SARS-CoV-2. NRP-1 is more abundant in the respiratory and olfactory epithelium, with the highest levels of expression in endothelium cells and nasal epithelial cells. NRPs are also used as an entrance factor by various viruses, including the human T cell lymphotropic virus type 1 (HTLV-1) cytomegalovirus (CMV), Epstein–Barr virus, and Lujo virus., and NRP-1 has been found to be an entrance factor for SARS-CoV-2 [32,33]. This is due to its location on epithelia that faces the external environment, allowing cells, arteries, and tissue to penetrate [34]. When comparing COVID-19 patients to healthy controls, the level of NRP-1 mRNA expression was found to be significantly higher in all severe and moderate COVID-19 patients. Furthermore, in our investigation, there was a strong association between NRP-1 mRNA expressions and mediators of inflammation, such as NF-κB p50, NF-κB p65, PGE2, and TXA2 in COVID-19 patients. Our findings demonstrated that two groups of COVID-19 patients had significant increases in four pro-inflammatory cytokines (IL-1β, IL-6, and IL-18) and two anti-inflammatory cytokines (IL-4 and IL-35). Several cytokines were shown to be significantly greater in the severe patients’ peripheral blood than in control patients [35]. COVID-19 symptoms include an uncontrolled and increased release of pro-inflammatory cytokines, as well as a weakened immune system, resulting in a cytokine storm. The uncontrolled and dysregulated release of inflammatory and pro-inflammatory cytokines is linked to the severity and mortality rate of viral infections. When compared to individuals who are moderately ill, critically ill patients have higher levels of pro-inflammatory cytokines including IL6 [10]. This conclusion is consistent with our findings, which showed that IL-6 levels were considerably greater in the severe patient group than in the moderate patient group. In the peripheral blood of COVID-19 patients, elevated IL-6 and IL-6-activated genes have been discovered [36]. Among a family of 20-carbon fatty acid derivatives identified together as eicosanoids, PGE2, and TXA2 are members of this family. They are responsible for normal physiology and inflammatory responses [37]. This study showed an increase in PGE2 and TXA2 in all severe and moderate patients compared with the healthy controls. These findings agree with Ricke-Hoch et al. [38], who reported that PGE2 is elevated in patients with COVID-19 disease. Elkhodary [39] reported that the overproduction of cytokines accompanies heavy infection of the virus. PGE2 aids IL-1β-dependent IL-6 synthesis from human fibroblasts via EP4 receptors in humans [40]. PGE2 increases the induction of IL-6 and other pro-inflammatory cytokines (e.g., IL-8) in monocytes, macrophages, fibroblasts, and airway epithelial cells in response to a variety of stimuli via both EP2 and EP4 receptors [41]. The catastrophic instances and deaths of COVID-19 are caused by cytokine overproduction (cytokine storms). 

The virus causes lymphopenia by stimulating immune cells such as macrophages to create two highly inflammatory cytokines, IL-1 and IL-6. IL-1 is the most common inflammatory cytokine, and once created it acts as an autocrine stimulator, encouraging the production of other cytokines as well as itself. Combining IL-1 and IL-6 in the liver exerts a synergistic pro-inflammatory effect on the production of serum amyloid A (SAA) [42]. IL-1 also stimulates other pro-inflammatory mediators, such as arachidonic acid molecules, which include TXA2. In addition to the cytokine storm, there is a storm of pro-inflammatory eicosanoids in SARS-CoV-2 infection, which includes the release of PGs, leukotrienes, and thromboxanes, which can help with pain, fever, pulmonary fibrosis, thrombosis, and ARDS [43]. Pro-inflammatory eicosanoids are produced mostly in the endothelium, which regulates vascular tone and promotes inflammation. As a result, eicosanoid inhibitors may be able to impact the inflammatory cascade and develop a viable therapeutic line in COVID-19 inflammation [43].

In the present study, NF-κB p50 and NF-κB p65 mRNA expression significantly increased in association with cytokine storm. In both severe and non-severe COVID-19 patients, NF-κB p50, NF-κB p65, and NRP-1 mRNA expression levels were linked with all cytokines. According to Notz et al. [44], 30.8% of the patients suffering from severe ARDS died. Every time point showed a significant increase in IL6. There was no discernible imbalance between pro- and anti-inflammatory pathways. Tan et al. [45] demonstrated that a general decrease in lymphocytes, as well as an increase in IL-6 and CRP, are valid indicators of severe COVID-19. In all COVID-19 instances, we found a considerable increase in ALT, AST, and CRP, as well as severe albumin depletion. ALT was found to be linked to IL-6. Infection with SARS-CoV-2 causes hyperactive immunological responses and systemic inflammation, as well as cytokine storms, which can disrupt several organs, including the gut and liver. Multiple organ failure is usually associated with the emergence of inflammatory reactions suddenly in critically ill COVID-19 patients [46]. IL-6 is a powerful stimulator of acute phase reactive protein, particularly serum amyloid A (SAA) and CRP [47], and can induce hepatocytes to synthesize acute phase reactive protein at the gene transcription level. The action of pro-inflammatory cytokines, including IL-1, IL-6, and IL-18, is ascribed to the clinical findings of cytokine storms [48]. In the cytokine storm, the Janus Kinase (JAK) and JAK/transcription factor 3 (STAT) signaling are critical; JAK/activator of STAT3 pathways can activate the NF-κB pathway. IL-6 stimulates the JAK-STAT pathway and phosphorylated STAT3, inducing cytokine release syndrome. NF-κB activation produces IL-6. The NF-κB and JAK-STAT pathways are activated during COVID-19, which can initiate the IL-6 amplifier response, resulting in NF-κB hyperactivation [11]. NF-κB was identified as a component of cytokine storm syndrome by Davies [49], and was linked to a higher severity of COVID-19-related symptoms. These findings are consistent with the findings of the current investigation, which indicated a significant rise in both NF-κB p50 and NF-κB p65 expression levels in severe and moderate patients when compared to healthy controls. Increased NF-κB p65-mediated transactivation plays a dangerous role in the aetiology of numerous chronic inflammatory illnesses, according to Giridharan and Srinivasan [50]. In severe COVID-19 cases, activated T lymphocytes target infected body cells, causing apoptosis and necrosis until T lymphocytes are exhausted, which is related to higher levels of Th17 and CD8 T cells, as well as IL-6 [35]. Numerous mechanisms have been hypothesized to generate anoxia. COVID-19’s trademark is respiratory failure, which is a credible cause of liver and organ morbidity. As a result, COVID-19-related problems might cause hypoxia and shock, which can lead to hepatic ischemia and hypoxia-reperfusion dysfunction [51]. Furthermore, ischemic/hypoxic liver injury is linked to metabolic acidosis and calcium excess, which are often shown by increased aminotransferase activity [52]. The large increase in ROS and peroxidation products in hypoxia can operate as a second messenger, boosting redox-sensitive transcription factors and increasing the production of several pro-inflammatory cytokines, culminating in liver damage. Specifically, during shock and hypoxic conditions, oxygen reduction and fat buildup in hepatocytes may contribute to hepatic cell death [51]. A straightforward and accurate biomarker for early COVID-19 disease in the emergency room prior to hospital admission would have a direct and immediate effect. To find new biomarkers that can be used in addition to RT-PCR or antigen assays, we analyzed the serum cytokine profile of COVID-19 patients. To demonstrate this, we have proven that TXA2 is statistically higher in moderate COVID-19 patients at hospital admission compared to severe COVID-19 patients, and IL-6 is higher in severe COVID-19 patients at hospital admission compared to moderate COVID-19 patients. ROC analysis revealed the significant probabilities of IL-6, TXA2, NF-κB p50 and NF-κB p65. TXA2 and NF-κB p65 had AUC higher than 0.8 in moderate COVID-19 patients, and IL-6 had AUC higher than 0.8 with a sensitivity of 100% in severe COVID-19 patients. Thus, these variables may be useful indicators to predict the possibility of COVID-19 infection, and IL-6 level may be a more reliable marker for the possibility of severity. These results are in accordance with Santa Cruz et al. [53] and El-Shabrawy et al. [54] who stated that the IL-6 level seems to be a valuable biomarker in evaluating the severity and progress of COVID-19 disease. 

## 5. Conclusions

The cytokine storm that causes COVID-19’s severity and mortality may be to blame for liver injury in COVID-19 patients. All cytokines in this study are linked to NF-κB p50 and NF-κB p65, as well as NRP-1 mRNA expression. As a result, COVID-19 can be suppressed by retaining NF-κB in an inactive state and suppressing NRP-1 expression. Moreover, ROC analysis in the study revealed that IL-6, TXA2 and NF-κB p65 may be of usefulness in predicting the probability of COVID-19 infection, and IL-6 in predicting the disease severity and progress. However, further clinical studies are necessary to confirm this finding. 

## Figures and Tables

**Table 1:** Comparison of Liver function tests; CRP; cytokine levels; PGE2; TXA2; NF-κB p50, NF-κB p65 and NRP-1 in the studied groups.

	Healthy (*n* = 50)	Moderate Patients (*n* = 50)	Severe Patients (*n* = 50)
ALT (U/I)	16.35 ± 0.71	98.44 ± 7.64 ***^,+++^	152.72 ± 11.37 ***
AST(U/I)	21.91 ± 1.00	83.54 ± 4.72 ***^,+^	100.82 ± 9.1 ***
Albumin (g/dl)	4.00 ± 0.06	3.80 ± 0.14 ***^,++^	3.06 ± 0.05 ***
CRP(mg/dl)	1.96 ± 0.20	51.75 ± 6.21 ***^.+++^	77.8 ± 3.33 ***
IL-1β (pg/mL)	15.20 ± 0.51	35.57 ± 1.88 ***^,+^	29.96 ± 2.06 ***
IL-4 (pg/mL)	4.07 ± 0.24	6.78 ± 0.27 ***	6.48 ± 0.44 ***
IL-6 (pg/mL)	10.37 ± 0.31	83.09 ± 5.25 ***^,+++^	110.37 ± 3.14 ***
IL-18 (pg/mL)	116.08 ± 0.96	217.64 ± 7.47 ***	229.68 ± 6.97 ***
IL-35 (pg/mL)	76.30 ± 1.39	106.03 ± 1.57 ***	105.05 ± 1.55 ***
PGE2(pg/mL)	136.93 ± 1.45	260.44 ± 8.55 ***	253.15 ± 8.78 ***
TXA2 (pg/mL)	114.96 ± 0.73	241.74 ± 7.02 ***^,+++^	211.75 ± 6.91 ***
NF-κB p50 expression	1.01 ± 0.002	2.29 ± 0.12 ***	2.28 ± 0.09 ***
NF-κB p65 expression	1.02 ± 0.003	4.38 ± 0.21 ***^,+^	3.89 ± 0.16 ***
NRP-1 expression	1.01 ± 0.002	4.28 ± 0.16 ***	3.98 ± 0.19 ***

Data are expressed as mean ± standard error (SE). Values were considered significantly different at *** *p* < 0.001 versus healthy control and ^+^ *p* < 0.05; ^++^ *p* < 0.01 and ^+++^ *p* < 0.001 versus severe group.

**Table 2:** Correlation between CRP, PGE2, TXA2, NF-κB p50, NF-κB p65 and NRP-1 with all parameters in the moderate group.

	CRP		PGE2		TXA2		NF-κB p50		NF-κB p65		NRP-1	
	R	*p*	R	*p*	R	*p*	R	*p*	R	*p*	R	*p*
ALT (U/L)	0.421 ***	0.000	0.303 **	0.002	0.407 ***	0.000	0.370 ***	0.000	0.362 ***	0.000	0.417 ***	0.000
AST(U/L)	0.477 ***	0.000	0.305 **	0.002	0.331 **	0.001	0.253 *	0.011	0.320 **	0.001	0.350 ***	0.000
Albumin (g/dL)	−0.646 ***	0.000	−0.162	0.106	0.001	0.994	−0.082	0.417	0.036	0.722	−0.161	0.110
CRP (mg/dL)	1		0.540 ***	0.000	0.515 ***	0.000	0.421 ***	0.000	0.461 ***	0.000	0.627 ***	0.000
IL-1β (pg/mL)	0.504 ***	0.000	0.464 ***	0.000	0.637 ***	0.000	0.481 ***	0.000	0.629 ***	0.000	0.604 ***	0.000
IL-4 (pg/mL)	0.375 ***	0.000	0.623 ***	0.000	0.681 ***	0.000	0.533 ***	0.000	0.463 ***	0.000	0.631 ***	0.000
IL-6 (pg/mL)	0.612 ***	0.000	0.749 ***	0.000	0.700 ***	0.000	0.572 ***	0.000	0.549 ***	0.000	0.817 ***	0.000
IL-18 (pg/mL)	0.634 ***	0.000	0.692 ***	0.000	0.718 ***	0.000	0.590 ***	0.000	0.611 ***	0.000	0.778 ***	0.000
IL-35 (pg/mL)	0.557 ***	0.000	0.624 ***	0.000	0.734 ***	0.000	0.619 ***	0.000	0.671 ***	0.000	0.775 ***	0.000
PGE2 (pg/mL)	0.540 ***	0.000	1		0.657 ***	0.000	0.487 ***	0.000	0.610 ***	0.000	0.711 ***	0.000
TXA2 (pg/mL)	0.515 ***	0.000	0.657 ***	0.000	1		0.773 ***	0.000	0.850 ***	0.000	0.835 ***	0.000
NF-κB p50 expression	0.421 ***	0.000	0.487 ***	0.000	0.773 ***	0.000	1		0.571 ***	0.000	0.726 ***	0.000
NF-κB p65 expression	0.461 ***	0.000	0.610 ***	0.000	0.850 ***	0.000	0.571 ***	0.000	1		0.706 ***	0.000
NRP-1 expression	0.627 ***	0.000	0.711 ***	0.000	0.835 ***	0.000	0.726 ***	0.000	0.706 ***	0.000	1	

Significance for correlation was calculated at three levels: * *p* < 0.05, ** *p* < 0.01, and *** *p* < 0.001.

**Table 3:** Correlation between IL1β, IL4, IL-6, IL-18, and IL-35 with all parameters in the moderate group.

	IL1β		IL4		IL6		IL18		IL35	
	R	*p*	R	*p*	R	*p*	R	*p*	R	*p*
ALT (U/L)	**R**	0.502	0.227 *	0.023	0.308 **	0.002	0.452 ***	0.000	0.328 **	0.001
AST(U/L)	0.068	0.541	0.218 *	0.029	0.318 **	0.001	0.460 ***	.000	0.332 **	0.001
Albumin (g/dL)	0.062	0.216	−0.025	0.808	−0.187	0.063	−0.218 *	0.029	−0.140	0.164
CRP(mg/dL)	−0.125	0.000	0.375 ***	0.000	0.612 ***	0.000	0.634 ***	0.000	0.557 ***	0.000
IL-1β (pg/mL)	0.504 ***		0.429 ***	0.000	0.530 ***	0.000	0.445 ***	0.000	0.610 ***	0.000
IL-4 (pg/mL)	1	0.000	1		0.513 ***	0.000	0.495 ***	0.000	0.592 ***	0.000
IL-6 (pg/mL)	0.429 ***	0.000	0.513 ***	0.000	1		0.793 ***	0.000	0.654 ***	0.000
IL-18 (pg/mL)	0.530 ***	0.000	0.495 ***	0.000	0.793 ***	0.000	1		0.606 ***	0.000
IL-35 (pg/mL)	0.445 ***	0.000	0.592 ***	0.000	0.654 ***	0.000	0.606 ***	0.000	1	
PGE2(pg/mL)	0.610 ***	0.000	0.623 ***	0.000	0.749 ***	0.000	0.692 ***	0.000	0.624 ***	0.000
TXA2 (pg/mL)	0.464 ***	0.000	0.681 ***	0.000	0.700 ***	0.000	0.718 ***	0.000	0.734 ***	0.000
NF-κB p50 expression	0.637 ***	0.000	0.533 ***	0.000	0.572 ***	0.000	0.590 ***	0.000	0.619 ***	0.000
NF-κB p65 expression	0.481 ***	0.000	0.463 ***	0.000	0.549 ***	0.000	0.611 ***	0.000	0.671 ***	0.000
NRP-1 expression	0.629 ***	0.000	0.631 **	0.000	0.817 ***	0.000	0.778 ***	0.000	0.775 ***	0.000

Significance for correlation was calculated at three levels: * *p* < 0.05, ** *p* < 0.01, and *** *p* < 0.001.

**Table 4:** Correlation between CRP, PGE2, TXA2, NF-κB p50, NF-κB p65 and NRP-1 with all parameters in the severe group.

	CRP		PGE2		TXA2		NF-κB p50		NF-κB p50		NRP-1	
	R	*p*	R	*p*	R	*p*	R	*p*	R	*p*	R	*p*
ALT (U/L)	0.401 ***	0.000	0.254 *	0.011	0.361 ***	0.000	0.299 **	0.002	0.423 ***	0.000	0.103	0.309
AST (U/L)	0.334 **	0.001	0.155	0.123	0.340 **	0.001	0.318 **	0.001	0.397 ***	0.000	0.092	0.361
Albumin (g/dL)	−0.722 ***	0.000	−0.599 ***	0.000	−0.625-***	0.000	−0.622 ***	0.000	−0.697 ***	0.000	−0.715 ***	0.000
CRP (mg/dL)	1		0.695 ***	0.000	0.733 ***	0.000	0.716 ***	0.000	0.834 ***	0.000	0.745 ***	0.000
IL-1β (pg/mL)	0.548 ***	0.000	0.265 **	0.008	0.530 ***	0.000	0.466 ***	0.000	0.390 ***	0.000	0.509 ***	0.000
IL4 (pg/mL)	0.400 ***	0.000	0.286 **	0.004	0.432 ***	0.000	0.193	0.055	0.293 ***	0.003	0.230 *	0.021
IL6 (pg/mL)	0.838 ***	0.000	0.799 ***	0.000	0.775 ***	0.000	0.773 ***	0.000	0.838 ***	0.000	0.811 ***	0.000
IL18 (pg/mL)	0.737 ***	0.000	0.754 ***	0.000	0.670 ***	0.000	0.707 ***	0.000	0.785 ***	0.000	0.731 ***	0.000
IL35 (pg/mL)	0.685 ***	0.000	0.640 ***	0.000	0.632 ***	0.000	0.728 ***	0.000	0.669 ***	0.000	0.708 ***	0.000
PGE2 (pg/mL)	0.695 ***	0.000	1		0.563 ***	0.000	0.707 ***	0.000	0.703 ***	0.000	0.677 ***	0.000
TXA2 (pg/mL)	0.733 ***	0.000	0.563 ***	0.000	1		0.619 ***	0.000	0.747 ***	0.000	0.576 ***	0.000
NF-κB p50 expression	0.716 ***	0.000	0.707 ***	0.000	0.619 ***	0.000	1		0.718 ***	0.000	0.735 ***	0.000
NF-κB p50 expression	0.834 ***	0.000	0.703 ***	0.000	0.747 ***	0.000	0.718 ***	0.000	1		0.657 ***	0.000
NRP-1 expression	0.745 ***	0.000	0.677 ***	0.000	0.576 ***	0.000	0.735 ***	0.000	0.657 ***	0.000	1	

Significance for correlation was calculated at three levels: * *p* < 0.05, ** *p* < 0.01, and *** *p* < 0.001.

**Table 5:** Correlation between IL1β, IL4, IL-6, IL-18, and IL-35 with all parameters in the severe group.

	IL1β		IL4		IL6		IL18		IL35	
	R	*p*	R	*p*	R	*p*	R	*p*	R	*p*
ALT (U/L)	0.268 **	0.007	0.313 **	0.002	0.288 **	0.004	0.258 **	0.009	0.199 *	0.047
AST (U/L)	0.203 *	0.043	0.204 *	0.042	0.286 **	0.004	0.232 *	0.020	0.200 *	0.046
Albumin (g/dL)	−0.495 ***	0.000	−0.330 **	0.001	−0.722 ***	0.000	−0.694 ***	0.000	−0.631 ***	0.000
CRP(mg/dL)	0.548 ***	0.000	0.400 ***	0.000	0.838 ***	0.000	0.737 ***	0.000	0.685 ***	0.000
IL-1β (pg/mL)	1		0.348 ***	0.000	0.544 ***	0.000	0.466 ***	0.000	0.450 ***	0.000
IL-4 (pg/mL)	0.348 ***	0.000	1		0.387 ***	0.000	0.341 **	0.001	0.425 ***	0.000
IL-6 (pg/mL)	0.544 ***	0.000	0.387 ***	0.000	1		0.805 ***	0.000	0.763 ***	0.000
IL-18 (pg/mL)	0.466 ***	0.000	0.341 **	0.001	0.805 ***	0.000	1		0.706 ***	0.000
IL-35 (pg/mL)	0.450 ***	0.000	0.425 ***	0.000	0.763 ***	0.000	0.706 ***	0.000	1	
PGE2 (pg/mL)	0.265 ***	0.008	0.286 **	0.004	0.799 ***	0.000	0.754 ***	0.000	0.640 ***	0.000
TXA2 (pg/mL)	0.530 ***	0.000	0.432 ***	0.000	0.775 ***	0.000	0.670 ***	0.000	0.632 ***	0.000
NF-κB p50 expression	0.466 ***	0.000	0.193	0.055	0.773 ***	0.000	0.707 ***	0.000	0.728 ***	0.000
NF-κB p65 expression	0.390 ***	0.000	0.293 **	0.003	0.838 ***	0.000	0.785 ***	0.000	0.669 ***	0.000
NRP-1 expression	0.509 ***	0.000	0.230 *	0.021	0.811 ***	0.000	0.731 ***	0.000	0.708 ***	0.000

Significance for correlation was calculated at three levels: * *p* < 0.05, ** *p* < 0.01, and *** *p* < 0.001.

**Table 6:** AUC, sensitivity and specificity calculation for IL-6, TXA2, NF-κB p50, and NF-κB p65 in moderate group.

	AUC	CI 95%	*p*	Cut-Off Value	Sensitivity	Specificity
IL-6 (pg/mL)	0.656	0.517–0.742	0.002	24.3 pg/ml	100%	50%
TXA2 (pg/mL)	0.843	0.782–0.904	<0.001	213.95 pg/ml	88%	75%
NF-κB p50 expression	0.735	0.657–0.814	<0.001	1.47 (relative to control)	80%	56%
NF-κB p65 expression	0.806	0.739–0.874	<0.001	3.35 (relative to control)	80%	67%

**Table 7:** AUC, sensitivity and specificity calculation for IL-6, TXA2, NF-κB p50, and NF-κB p65 in severe group.

	AUC	CI 95%	*p*	Cut-Off Value	Sensitivity	Specificity
IL-6 (pg/mL)	0.844	0.783–0.904	<0.001	51.0 pg/ml	100%	68%
TXA2 (pg/mL)	0.657	0.571–0.742	0.002	148.7 pg/ml	92%	53%
NF-κB p50 expression	0.765	0.690–0.839	<0.001	1.84 (relative to control)	74%	74%
NF-κB p65 expression	0.694	0.612–0.776	<0.001	3.25 (relative to control)	70%	60%

## Data Availability

The data are contained within the article.
